# Integrated Transcriptomic Analysis Reveals a Distinctive Role of YAP1 in Extramedullary Invasion and Therapeutic Sensitivity of Multiple Myeloma

**DOI:** 10.3389/fonc.2021.787814

**Published:** 2022-01-04

**Authors:** Bo Zheng, Wei Sun, Ke Yi, Yajun Zhang, Liangzhe Wang, Hongyan Lan, Chong Zhang, Hongming Xian, Rong Li

**Affiliations:** Nuclear Radiation Injury Protection and Treatment Department, Navy Medical Center of People Liberation Army (PLA), Naval Medical University, Shanghai, China

**Keywords:** YAP1, multiple myeloma, extramedullary invasion, therapy, bioinformatics

## Abstract

Multiple myeloma (MM) is the second most common hematologic malignancy. There are no standard therapeutic guidelines for extramedullary invasion (EM). We performed a retrospective integrated transcriptomic analysis based on GEO, TCGA, and Oncomine datasets with a total of over 2,500 cases enrolled. GSVA analysis was performed on GSE24080. The external validation cohorts include GSE9782, GSE2658, MMRF-COMPASS, and Oncomine. The data of MGUS to relapsed MM were acquired from GSE6477, GSE5900, and Oncomine. The data of EM were acquired from GSE39683 and GSE66291. Single-cell level transcriptome data of MM and EM were acquired from GSE106218. GSVA analysis revealed that 559 cases could be divided into 2 groups based on the expression of oncogenic pathways with prognostic significances. Group 1 with a specific phenotype of YAP1-MYC+ exhibited an unpromising prognosis. The univariate analysis revealed YAP1 as a tumor suppressor in MM. The activity of DNA repair, glycolysis, and oxidative phosphorylation was significantly higher in YAP1-MYC+ MM, which is in concordance with EM myeloma cells based on single-cell analysis. Furthermore, we discovered that YAP1-MYC+ MM patients exhibited an improved response for IMiD treatment. Collectively, YAP1-MYC+MM patients might suffer a worse prognosis and stronger propensity for EM progression.

## Introduction

Multiple myeloma is a malignant plasma cell disease with an aberrant proliferation of mature B cells. MM accounts for 1.8% of all malignancies and is the second most common hematologic malignancy (1, 2). The diagnosis of MM is defined by the presence of ≥ 10% clonal plasma cells in the bone marrow (3). The development of MM is usually complicated with end-organ damage, which is manifested by renal failure, anemia, bone lesions, and hypercalcemia (1). Since the introduction of novel agents including immunomodulators (IMiDs), proteasome inhibitors (PI), monoclonal antibodies, and histone deacetylating agents, the prognosis of MM patients has been significantly improved (4). However, the therapeutic responses and survival time of newly diagnosed MM patients differ from 2 to >10 years (5).

The development of MM originates from monoclonal gammopathy of undetermined significance (MGUS) and eventually progresses to extramedullary disease, which indicates that malignant plasma cells migrate beyond the restriction of bone marrow. Besides, there are no current guidelines regarding EM treatment. Therefore, the occurrence of EM significantly impaired the prognosis of MM patients (6). It is of urgent need to elucidate the molecular mechanism behind extramedullary invasion and identify possible therapeutic targets.

Gene chips have been widely applied as a gene detection technology in MM and corresponding data are deposited in multiple public online datasets. Integrating and reanalyzing these genomic data offer possibilities for identifying novel molecular mechanisms and therapeutic targets. However, the conclusions of previous bioinformatic publications are mostly limited by a lack of clinical relevance and external validation.

In this study, we collected transcriptome data of over 2,500 MM cases from multiple public sources including the NCBI‐Gene Expression Omnibus database (NCBI‐GEO), the Cancer Genome Atlas (TCGA), and Oncomine database and performed a retrospective *in silico* analysis. We performed GSVA analysis on 559 MM cases from GSE24080. The external validation cohort includes over 2,000 MM cases from GEO datasets (GSE9782, GSE2658), the TCGA dataset (MMRF-COMPASS), and Oncomine dataset (Zhan myeloma 2). The treatment information was acquired from MMRF-COMPASS. To explore the molecular mechanism in MM development, we also enrolled GSE6477 and Oncomine (Agnelli myeloma 3). GSE39683 and GSE66291 were enrolled to study the transcriptomic differences between primary MM and EM. Single-cell level transcriptome data were acquired from GSE106218 to analyze the transcriptomic evolution in MM and EM myeloma cells.

Collectively, we performed a retrospective multi-center, integrated transcriptomic analysis on both bulk and single-cell level in MM. This study aims to identify novel molecular mechanisms behind extramedullary invasion and therapeutic responses and hopefully provide new therapeutic targets in MM and EM.

## Materials and Methods

### Data Collections and Availability

The microarray mRNA expression profiles and related clinical information for GSVA analysis were obtained from GSE24080 on the GEO database (https://www.ncbi.nlm.nih.gov/geo/). External validation GEO datasets include GSE7982 and GSE2658. The transcriptome of different developmental stages of MM was obtained from GSE6477. Transcriptome data of sPCL samples were obtained from GSE39683 and GSE66291. The TCGA-MMRF-COMPASS project (https://portal.gdc.cancer.gov/projects) consisted of 787 MM transcriptome data along with corresponding clinical information. Oncomine datasets (https://www.oncomine.org/resource/login.html) include Agnelli myeloma 3, Zhan myeloma 2. The single-cell transcriptome data were obtained from GSE106218. The expression matrix of GEO, TCGA datasets along with detailed clinical characteristics of COMPASS-MMRF patients used in this study can be accessed and cited *via Zheng, Bo (2021), “The distinctive role of YAP1 in multiple myeloma”, Mendeley Data, V2, doi: 10.17632/mmp7cw7rx9.2.*

### GSVA Analysis

The GSVA software package (V1.25.4) for R was applied as a non-parametric, unsupervised method for estimating the variation of key gene sets in MM. The input for the GSVA algorithm was a gene expression matrix of log2 microarray expression values and a collection of *C6-oncogenic signature gene sets* from the Molecular Signature Database (MSigDB, version 7.4). GSVA scores were determined nonparametrically using a Kolmogorov–Smirnov (KS)-like random walk statistic and a negative value for a particular sample and gene set.

### Survival Analysis and Univariate Analysis

The “survival” and “survminer” R packages were applied for survival analysis. Surv_cutpoint function was applied to acquire the optimal cutoff value. The survival information of GEO and TCGA datasets was acquired along with their expression matrix data. *p*-value less than 0.05 was considered significant. A single-factor Cox model was used to determine whether a single gene was related to the prognosis of MM.

### Reactome Enrichment Analysis

The “Clusterprofiler” package (V3.16.0) of R language was applied for enrichment analysis of Reactome pathways. “Clusterprofiler” is an R package of Bioconductor, which can perform statistical analysis and visualization of functional clustering on gene sets or gene clusters. When the adjusted *p*-value was below 0.05, the Reactome pathways were identified as significantly enriched by these genes.

### GSEA Analysis

To unveil biological correlations of the obtained gene expression profiles, the transcriptome data were compared using GSEA (http://www.gsea-msigdb.org/gsea/). GSEA uses a weighted Kolmogorov–Smirnov method to determine whether the distribution of genes in the gene set is different from the normal distribution. h.all.v7.4.symbols and c2.cp.reactome.v7.4.symbols were selected for analysis. False discovery rate (FDR) < 0.05 and adjusted *p*-value < 0.05 were considered statistically significant.

### Trajectory and Pseudotime Analysis

“Monocle2 (v2.16.0)”, an R package, was applied to conduct single-cell evolutionary trajectory analysis and to estimate the transcriptome evolution in primary and extramedullary MM samples. Monocle is based on the assumption that one-dimensional “time” can depict the multi-dimensional expression values to elucidate the cell state transitions. In the trajectory analysis, we used genes meeting the following standards: mean_expression ≥ 0.1 and dispersion_empirical ≥ 1 * dispersion_fit to sort cells in pseudo-time order. The visualization functions “plot_cell_trajectory” were used to plot the minimum spanning tree on cells. Genes that changed along with the pseudotime were calculated (*q*-value <0.01) by the “differentialGeneTest” function and visualized with the plot_pseudotime_heatmap and the genes were clustered into subgroups according to the gene expression patterns.

### Statistical Analysis

All statistical analyses were performed using R 4.1.0 and GraphPad prism 8. Two-sided Student’s *t*-test for unpaired samples was applied to evaluate the significance of differences in experiments. Pearson correlation coefficients were calculated to measure associations among the mRNA expression level of various genes. OS and RFS were assessed with the Kaplan–Meier method, and the differences between the groups were compared by the log-rank test. Values of *p* and *q* less than 0.05 were considered statistically significant. Other key packages used in this study include *GGally*, *ggrisk*.

## Results

### Gene Set Variation Analysis of 559 Newly Diagnosed MM Cases

**Figure 1:**
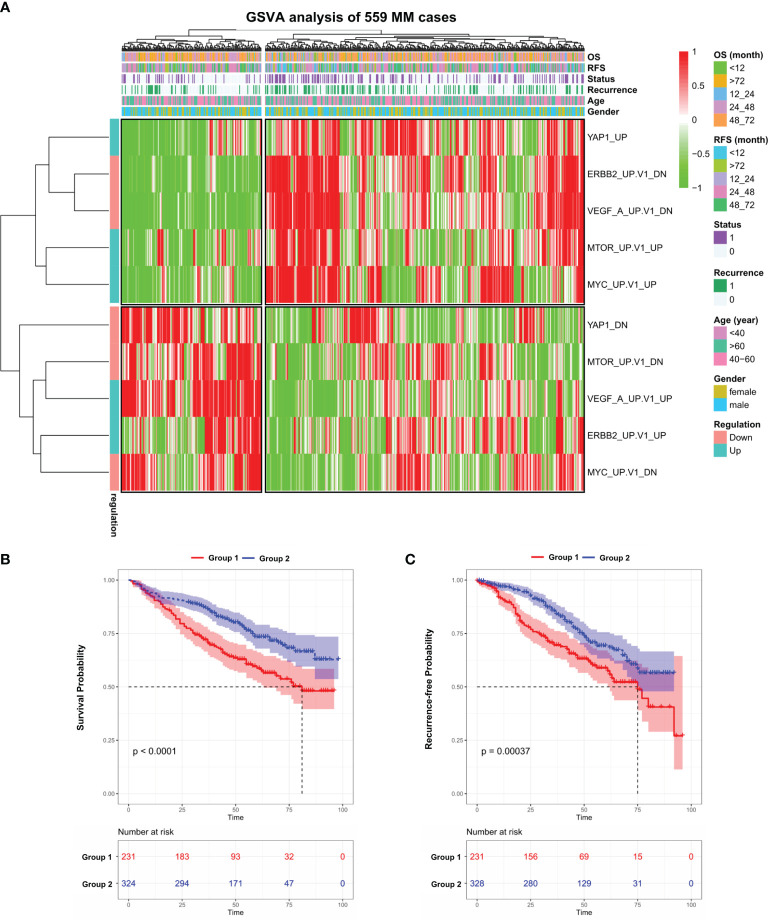
Gene set variation analysis of 559 newly diagnosed MM cases. **(A)** Heatmap of gene set variation analysis (GSVA) of 559 Multiple Myeloma transcriptome data (GSE24080) based on 10 selected *C6-oncogenic signature gene sets* from Molecular Signature Database. The relevant clinical information was added above. **(B)** Kaplan–Meier survival curves comparing the overall survival in MM group 1 and 2 from the GSE24080 dataset. **(C)** Kaplan–Meier survival curves comparing the recurrence-free survival in MM group 1 and 2 from the GSE24080 dataset. Results are shown as mean ± CI based on the log-rank *t*-test.

We performed GSVA analysis on the GEO dataset (*GSE24080*), which contains the transcriptome data of bone marrow purified plasma cells from 559 newly diagnosed MM patients. The median age of this cohort is 58 and male accounts for 60.3% (**Table S1**). GSVA analysis aims to investigate whether a set of genes are randomly distributed in a specific phenotype compared with others and identifies biological or pathological correlations. To this end, we examined the expression of *C6-oncogenic signature gene sets* from the Molecular Signature Database (MSigDB, version 7.4) in all 559 MM cases. The result showed that 599 patients were separated into two groups (**Figure S1A**). We further identified 5 signaling pathways that fit the following criteria: significantly up/downregulated in one MM Group while exhibiting the opposite in the other group. In the heatmap (**Figure 1A** and **Table S2**), we observed that in Group 1, which contains 231 patients, VEGFA and ERBB2 pathways were activated while other oncogenic pathways such as MYC, mTOR, and YAP1 exhibited the opposite. This unusual phenomenon might be attributed to the different molecular landscapes between solid and blood cancers. Notably, patients in Group 1 suffered from a significantly shorter survival (*p* < 0.0001) and higher recurrence rate (*p* < 0.001) compared with Group 2 (**Figures 1B, C**). Next, we investigated the expression level of the above 5 key genes in the pan-cancer scenario in *TIMER* database and found that YAP1 was the only gene significantly downregulated in blood cancers while consistently highly expressed in solid tumors (**Figure S1B**).

### The Correlation Among 5 Pathways Dysregulated in MM Patients and Their Prognostic Significances

**Figure 2:**
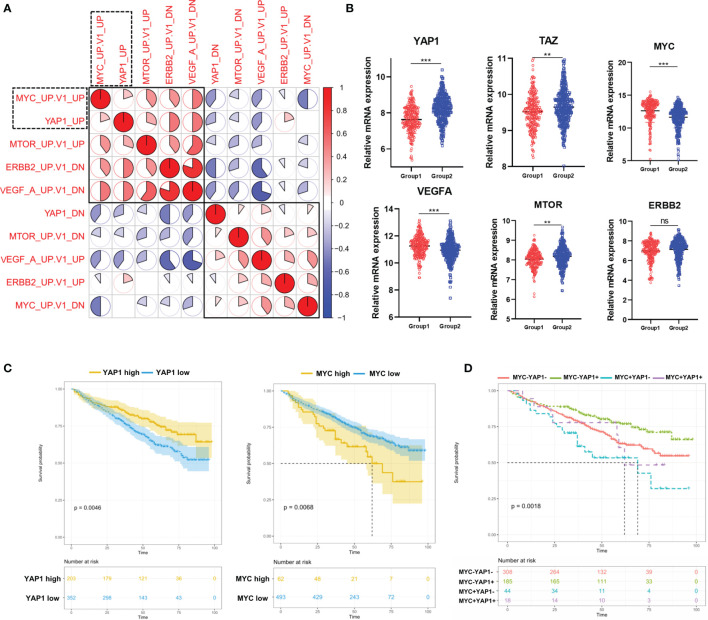
The correlation among 5 pathways dysregulated in MM patients and their prognostic significances. **(A)** Heatmap of the correlation among 5 cancer-related signaling pathways using Pearson correlation coefficients. **(B)** The comparison of mRNA expression level of YAP1/MYC/MTOR/ERBB2/VEGFA/TAZ in 2 MM groups. Results are shown as mean ± SD. *p < 0.05, **p < 0.01, ***p < 0.001, based on the Student’s *t*-test. **(C)** Kaplan–Meier survival curves comparing the overall survival in MM cases with high or low expression of YAP1/MYC in the GSE24080 dataset. **(D)** Kaplan–Meier survival curves comparing the overall survival in MM cases with differential expression levels of YAP1 and MYC in the GSE24080 dataset. ns, not significant.

Considering that the above 4 oncogenic pathways were specifically dysregulated along with YAP1, we moved on to investigate the correlation among them. The result showed that the YAP1 pathway was positively correlated with MYC and mTOR pathways while negatively correlated with VEGFA and ERBB2 pathways (**Figure 2A**). The Hippo/YAP1/TAZ pathway is well known as being highly conservative and the activation of the YAP1/TAZ complex is responsible for cell proliferation and anti-apoptosis (7). In solid tumors, the YAP1/TAZ complex improves the transcription of a series of oncogenes including MYC (8). In our study, YAP1 and TAZ were downregulated in Group 1 while MYC along with VEGFA and mTOR exhibited the opposite (**Figure 2B**). YAP1 showed a weak while statistically significant negative correlation with MYC and VEGFA (**Figure S2A**). Furthermore, YAP1 exhibited a positive prognostic factor in MM while MYC was a negative prognostic factor (**Figure 2C**). YAP1+MYC- patients showed the most favorable prognosis while YAP1-MYC+ patients showed the worst prognosis (**Figure 2D**). TAZ, VEGAF, and ERBB2 were all identified as negative prognostic factors in MM patients (**Figure S2B**).

### External Validation of the Prognostic Significance of YAP1/MYC and Their Impact on MM Therapies

**Figure 3:**
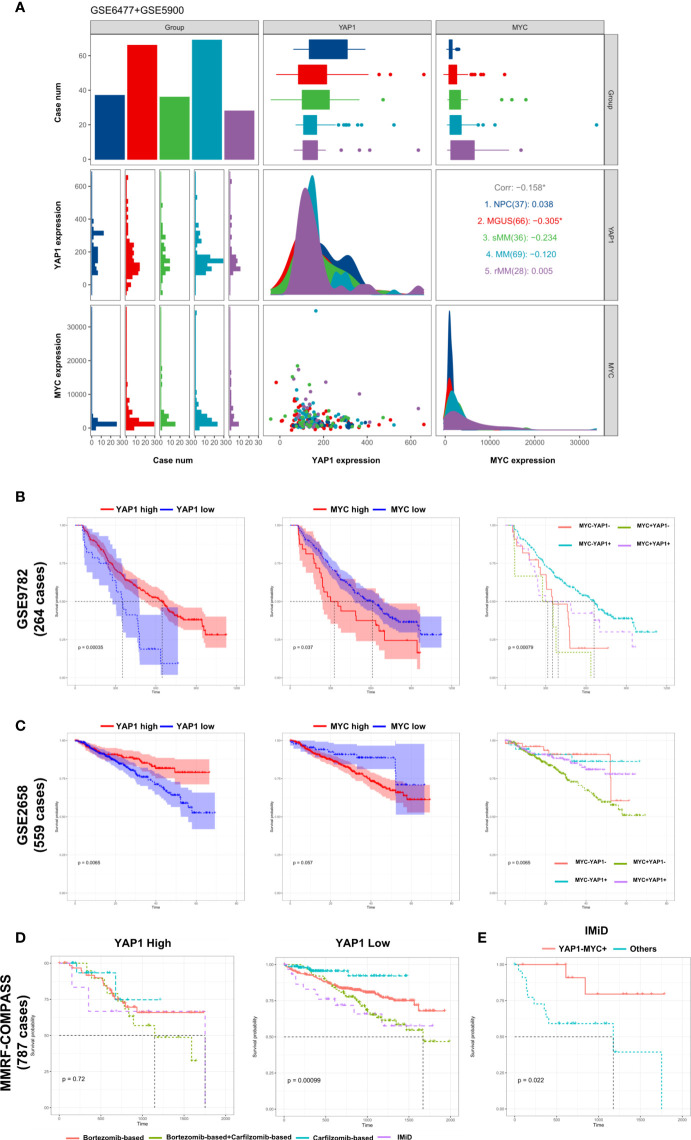
External validation of the prognostic significance of YAP1/MYC and their impact in MM therapies. **(A)** The comparison of YAP1/MYC mRNA expression levels in different developmental stages of MM. The *x-*axis represents case number/YAP1 count value/MYC count value from left to right while the *y*-axis represents case number/YAP1 count value/MYC count value from top to bottom. NPC: normal plasma cell; MGUS: monoclonal gammopathy of undetermined significance; sMM: smothering multiple myeloma; rMM: relapsed multiple myeloma. **(B)** Kaplan–Meier survival curves comparing the overall survival in MM cases with differential expression level of YAP1 and MYC in GSE9782 dataset (264 cases). **(C)** Kaplan–Meier survival curves comparing the overall survival in MM cases with differential expression level of YAP1 and MYC in the GSE2658 dataset (559 cases). **(D)** The survival rate of MM patients with high or low expression of YAP1 treated with different strategies. **(E)** The survival rate of YAP1-MYC+ MM patients versus others treated with IMiD.

The development of multiple myeloma goes through monoclonal gammopathy of undetermined significance (MGUS), smoldering multiple myeloma (sMM), symptomatic MM, and relapsed MM. Based on the data from GSE6477 and GSE5900 (9), we discovered that the expression of YAP1 decreased gradually from NC to sMM until a quick drop is experienced when progressing into MM. This phenomenon showed that YAP1 level was negatively correlated with tumor burden in MM. Comparably, MYC exhibited a gradual increase from NC to relapsed MM cases (**Figure 3A**). The same trend was validated in an external Oncomine dataset (*Angelli Myeloma 3*) with an exception of normal plasma cells probably due to fewer cases enrolled (**Figure S3B**). The prognostic significances of YAP1/MYC were further confirmed in 4 external independent datasets including GEO datasets (*GSE9782* and *GSE2658*), TCGA dataset (*MMRF-COMPASS*), and Oncomine dataset (*Zhan myeloma 2*) in over 2,000 MM patients (**Figures 3B, C** and **Figures S3C, D**). Treatment information of 787 MM patients was acquired from the MMRF-COMPASS study (**Table S3**). Notably, the expression level of YAP1 efficiently affected the response of various treatments. In MM patients with low YAP1 expression, the carfilzomib-based treatment showed obvious superiority over other treatments compared with other MM patients (**Figure 3D**). In MM patients with high MYC expression, the efficacy of IMiD treatment was improved compared with MM patients with low MYC expression (**Figure S3E**) and this trend was further amplified when we compared YAP-MYC+ MM patients with the rest (**Figure 3E** and **Figure S3F**).

### Aberrant Biological Processes in YAP1-Low Expressed MM Cases Enhanced Its Oncogenicity

**Figure 4:**
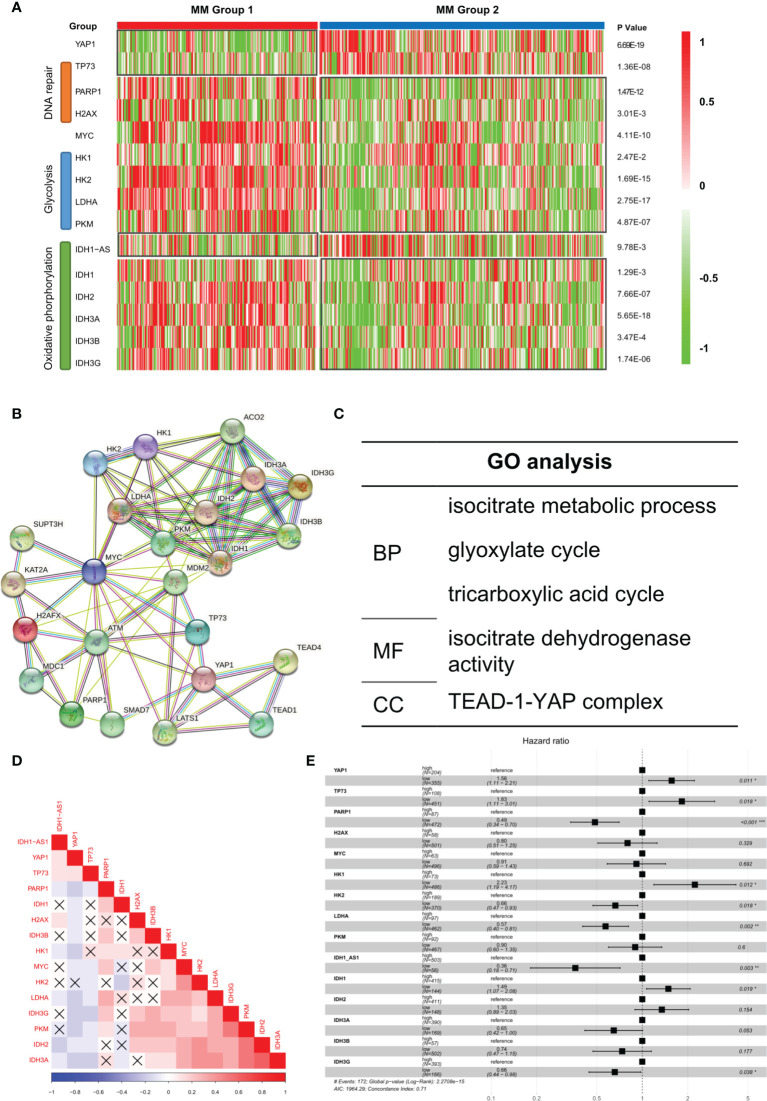
Aberrant biological processes in YAP1-low expressed MM cases enhanced its oncogenicity. **(A)** Heatmap of the expression level of YAP1/MYC along with DNA repair/glycolysis/oxidative phosphorylation-related genes in MM group 1 and 2. **(B, C)** Protein–protein interaction network of YAP1/MYC along with DNA repair/Glycolysis/Oxidative phosphorylation-related genes based on the STRING online database and GO term enrichment analysis. **(D)** Heatmap of the correlation of YAP1/MYC along with DNA repair/glycolysis/oxidative phosphorylation-related genes. *p*-value > 0.05 was annotated with an error mark. **(E)** Forest plot shows the univariate result of YAP1/MYC along with DNA repair/glycolysis/oxidative phosphorylation-related genes in MM.

It was reported that low YAP1 level in hematological cancers resulted in an improved DNA repairing process, which facilitated cell proliferation and improved oncogenicity (9). We observed that DNA repair-related genes such as PARP1 and H2AX were improved in Group 1 while apoptosis-related gene TP73 was suppressed (**Figure 4A**). According to previous studies, in extramedullary myeloma cells, the glycolysis process and oxidative phosphorylation process were enhanced (10). We found that glycolysis-related genes and oxidative phosphorylation-related genes were significantly upregulated in Group 1 patients. Based on the STRING database, the protein–protein interaction (PPI) network of the genes in **Figure 4A** was drawn (**Figure 4B**). The GO analysis result showed that multiple key energy pathways were involved (**Figure 4C**), indicating a metabolic reprogramming in Group 1 patients, which might lead to its increased oncogenicity and unpromising prognosis. In the expression correlation heatmap, we observed that in Group 1 patients, metabolic genes were mostly co-expressed (**Figure 4D**). The univariate analysis result showed that most metabolic genes posed as oncogenes while YAP1 and TP73 posed as tumor suppressor genes (**Figure 4E**). In GSE39683 and GSE66291 datasets, we acquired the transcriptome data of MM and second plasma cell leukemia (sPCL) and found that YAP1 was also downregulated in sPCL while MYC showed no difference (**Figure S4A**). With further exploration, we discovered that the activity of DNA repair, glycolysis, and oxidative phosphorylation was enhanced in sPCL, which simulated the phenotype of YAP1-MYC+ MM (**Figure S4B**), indicating that lower YAP1 expression might facilitate extramedullary invasion.

### Dysregulated DNA Repair Process and Metabolic Reprogramming in Group 1 MM Facilitated Extramedullary Invasion

**Figure 5:**
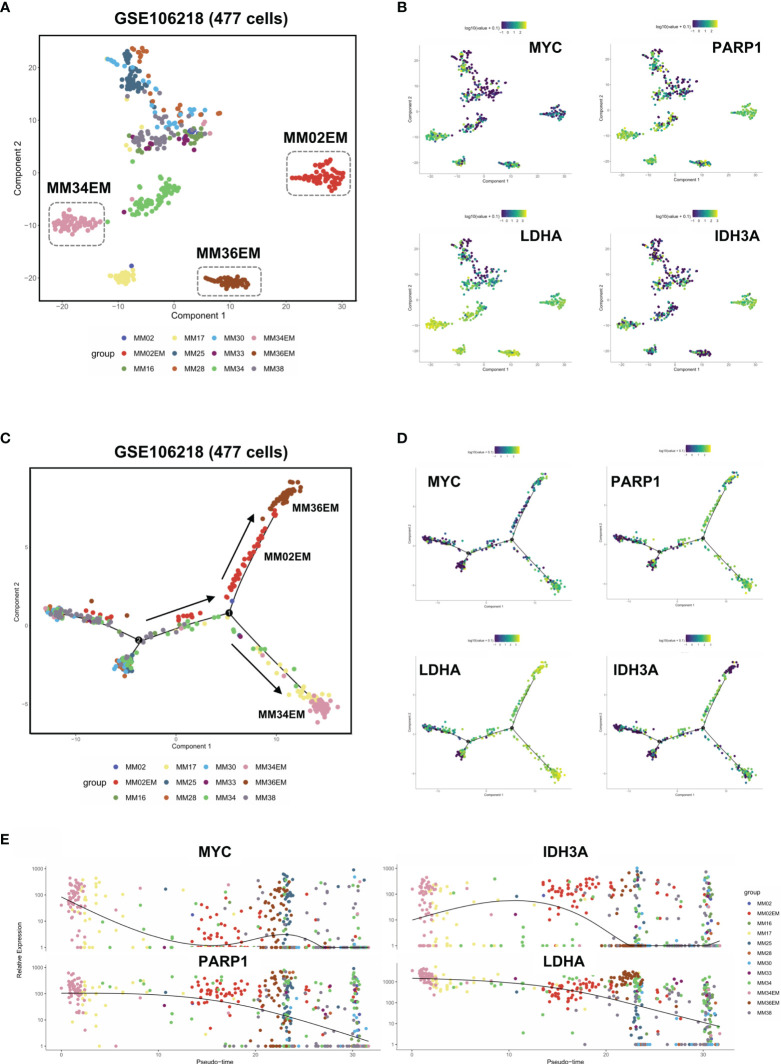
Dysregulated DNA repair process and metabolic reprogramming in Group 1 MM facilitated extramedullary invasion. **(A)** tSNE plots of 447 single-cell transcriptome data of primary and extramedullary MM samples based on the GSE106218 dataset. **(B)** The distribution and intensity of the expression of MYC/PARP1/LDHA/IDH3A on tSNE plots. **(C)** Pseudo time analysis of 447 single-cell transcriptome data *via* Monocle algorithm. Arrows indicated evolutionary directions. **(D)** The distribution and intensity of the expression of MYC/PARP1/LDHA/IDH3A on Monocle plots. **(E)** The expression intensity of MYC/PARP1/LDHA/IDH3A along pseudo time axis.

To further analyze the intratumoral heterogeneity in terms of YAP1/MYC expression, we collected the single-cell transcriptome data of 477 myeloma cells from GSE106218 which contained 9 MM samples and 3 paired EM samples. However, YAP1 data were not available on the single-cell level due to its low expression. Hence, we focus on the role of MYC in the process of extramedullary invasion. First, we performed dimension reduction analysis and displayed the sc-seq data on tSNE to investigate the similarities and divergence among different clusters and samples (**Figure 5A**). The result showed that 3 EM samples were well isolated from the clusters of primary MM samples except for MM17. We found that DNA repair and metabolic genes were marked upregulated in EM cases along with MM17 (**Figure 5B**). To examine the evolutionary transcriptomic change between primary MM and extramedullary myeloma, we performed a trajectory analysis for all 12 samples. Pseudotime analysis indicated a branched evolution from primary myelomas to extramedullary myelomas (**Figure 5C** and **Figure S5A**). Intriguingly, along the two extramedullary branches, MM02EM and MM36EM stayed together while MM34EM was located on the other branch. Comparably, MM34EM expressed the highest level of DNA repair and metabolic genes along with MYC (**Figure 5B**). Also, the MM34EM case experienced the shortest extramedullary invasion time (3 months) compared with MM02EM and MM36EM (both 20 months) (**Table S4**). MM17, which showed a similar phenotype as EM samples (**Figure 5B**), is located on the same extramedullary branch with MM34EM, indicating a propensity for EM invasion (**Figure 5C**). Notably, MM17 had the shortest survival time (3 months) among all primary MM cases. The expressions of DNA repair and metabolic genes along with MYC were marked enhanced on the extramedullary branches and experienced a downfall from EM to MM cases (**Figures 5D, E**)

### Differentially Expressed Genes between Group 1 and Group 2 in GSE24080

**Figure 6:**
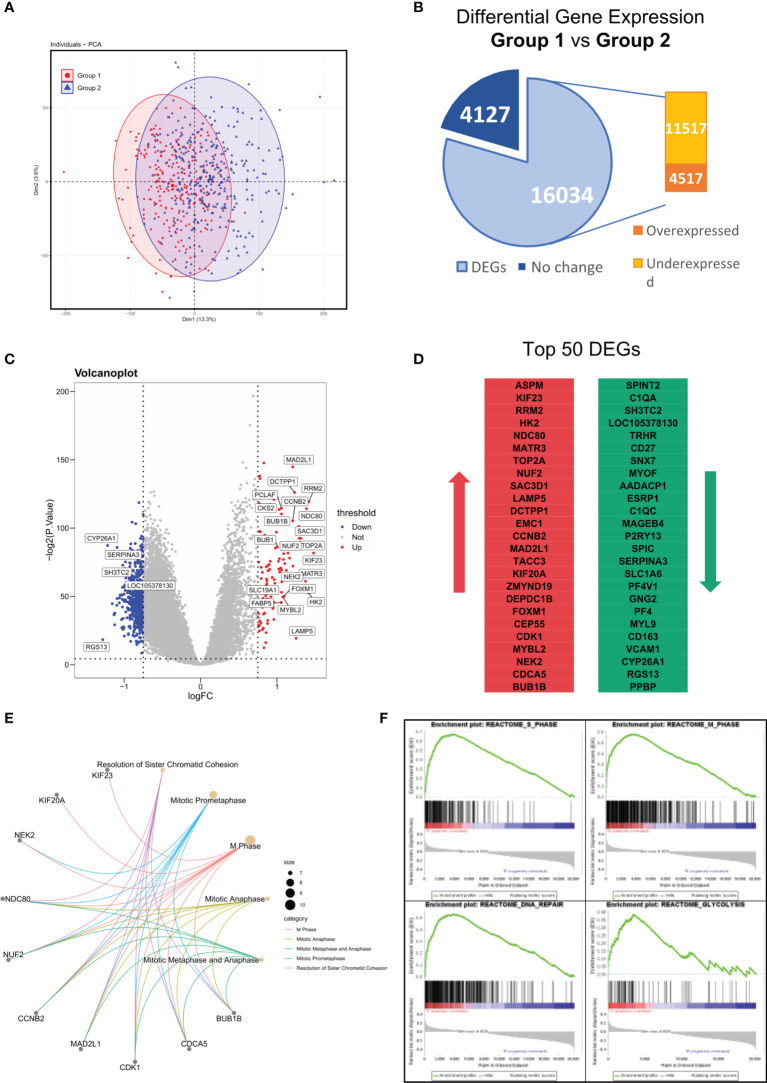
Differentially expressed genes (DEG) between Group 1 and Group 2 in GSE24080. **(A)** Principal components analysis of transcriptome data of group 1/2 MM patients from the GSE24080 dataset. **(B)** Differential gene expressions of group 1 relative to group 2 MM samples. Genes with positive log2 FC and *p* < 0.05 were considered overexpressed, and genes with negative log2 FC and *p* < 0.05 were considered underexpressed. **(C)** Volcano plots of DEGs. Red/Blue plots represent genes with |log2 FC| > 1 and *p* < 0.05. **(D)** List of the 50 most deregulated genes: the 25 most overexpressed and the 25 most underexpressed genes in group 1 relative to group 2 MM samples. **(E)** The top 5 reactome pathways enriched in the top 50 DEGs between groups 1 and 2. **(F)** The GSEA analysis of reactome pathways upregulated in group 1 MM patients.

In the results above, we identified a MM subgroup with a unique molecular feature, prognostic significance, and therapeutic response. Hence, we next intended to explore deeper into the difference of its mRNA expression pattern. To investigate the transcriptome signature of Group 1 (YAP1-MYC+) and Group 2 (YAP1+MYC-) cases, we firstly explored the distribution of all samples based on similarities in gene expression data. The PCA analysis revealed huge nonoverlapping areas between the 2 groups (**Figure 6A**). Gene expression analysis identified a total of 16,034 genes differentially expressed between two groups, of which 4,517 were overexpressed and 11,517 genes were underexpressed in Group 1 relative to Group 2 patients (**Figure 6B** and **Table S5**). Exemplary DEGs were annotated in the volcano plot and heatmap (**Figure 6C** and **Figure S6A**). The list of the 50 genes with the largest and smallest fold change (FC) is shown in **Figure 6D**. Functional analysis using the Reactome database revealed statistically significant enrichment for mitotic prometaphase, M phase, mitotic anaphase, mitotic metaphase/anaphase, etc. (**Figure 6E** and **Figure S6B**, **Table S6**). Since the functional analysis in **Figure 6E** did not take the fold change of gene expression into account, we performed GSEA analysis to identify the key changes in biological processes in terms of reactome database and Hallmark gene sets (MSigDB, version 7.4) between Group 1 and Group 2 (**Tables S7**, **S8**). The result showed that cell cycle was accelerated in Group 1 patients along with enhanced glycolysis, DNA repair, and oxidative phosphorylation processes. These findings confirmed that the improved oncogenicity in Group 1 MM cases resulted in high cellular proliferation (**Figure 6F** and **Figure S6C**). Additionally, we discovered that MM patients with the presence of soft tissue plasmacytoma exhibited enhanced activity of enhanced glycolysis, DNA repair, and oxidative phosphorylation processes along with mitosis (**Figure S6D**). Also, a subgroup of MMRF patients with low YAP1 expression and high MYC expression exhibited stronger activities of DNA repair/Glycolysis/Oxidative phosphorylation (**Figure S6E**).

### Dysregulated Biological Processes in Group 1 MM Cases Shorten Survival and Affect Therapeutic Responses

**Figure 7:**
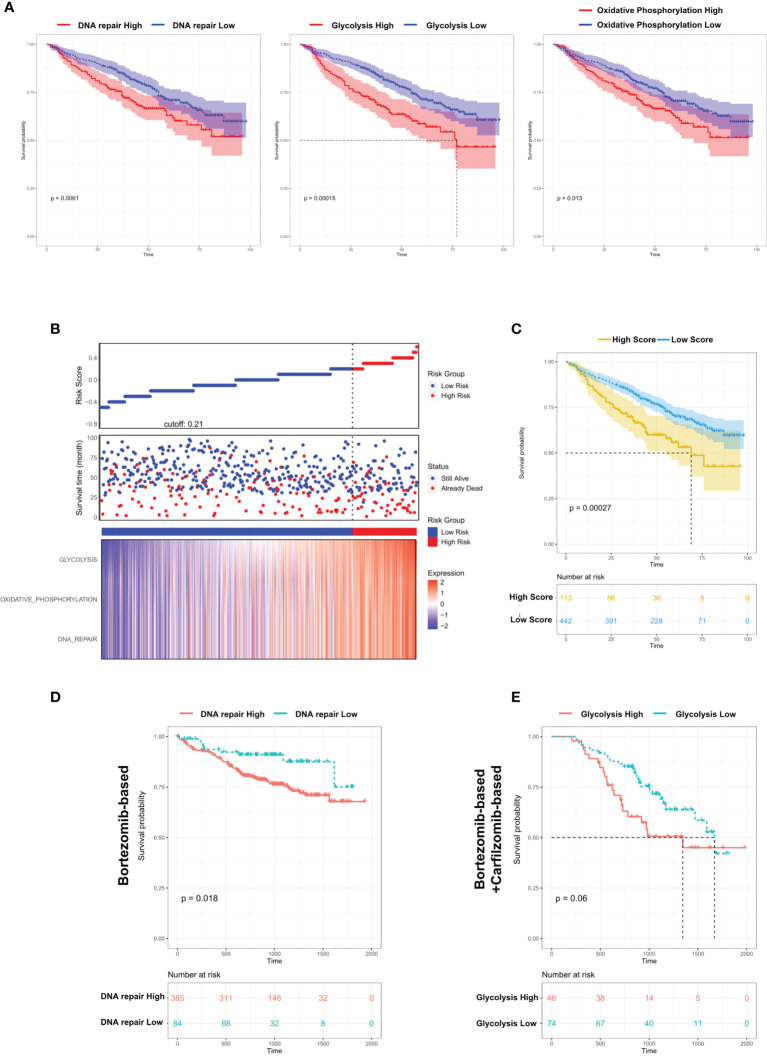
Dysregulated biological processes in Group 1 MM cases shorten survival and affect therapeutic responses. **(A)** Kaplan–Meier survival curves comparing the overall survival in MM cases with high or low expression of DNA repair/glycolysis/oxidative phosphorylation scores in the GSE24080 dataset. **(B)** Distribution of risk score, OS, survival status (red dots indicate dead; green dots indicate alive), and the three pathway scores’ heatmap in GSE24080. **(C)** Kaplan–Meier survival curves comparing the overall survival in MM cases with high- or low-risk score in the GSE24080 dataset. **(D)** The survival rate of MM patients with high/low DNA repair score treated with Bortezomib-based therapy. **(E)** The survival rate of MM patients with high/low glycolysis score treated with Bortezomib-based+Carfilzomib-based therapy.

**Figure 8:**
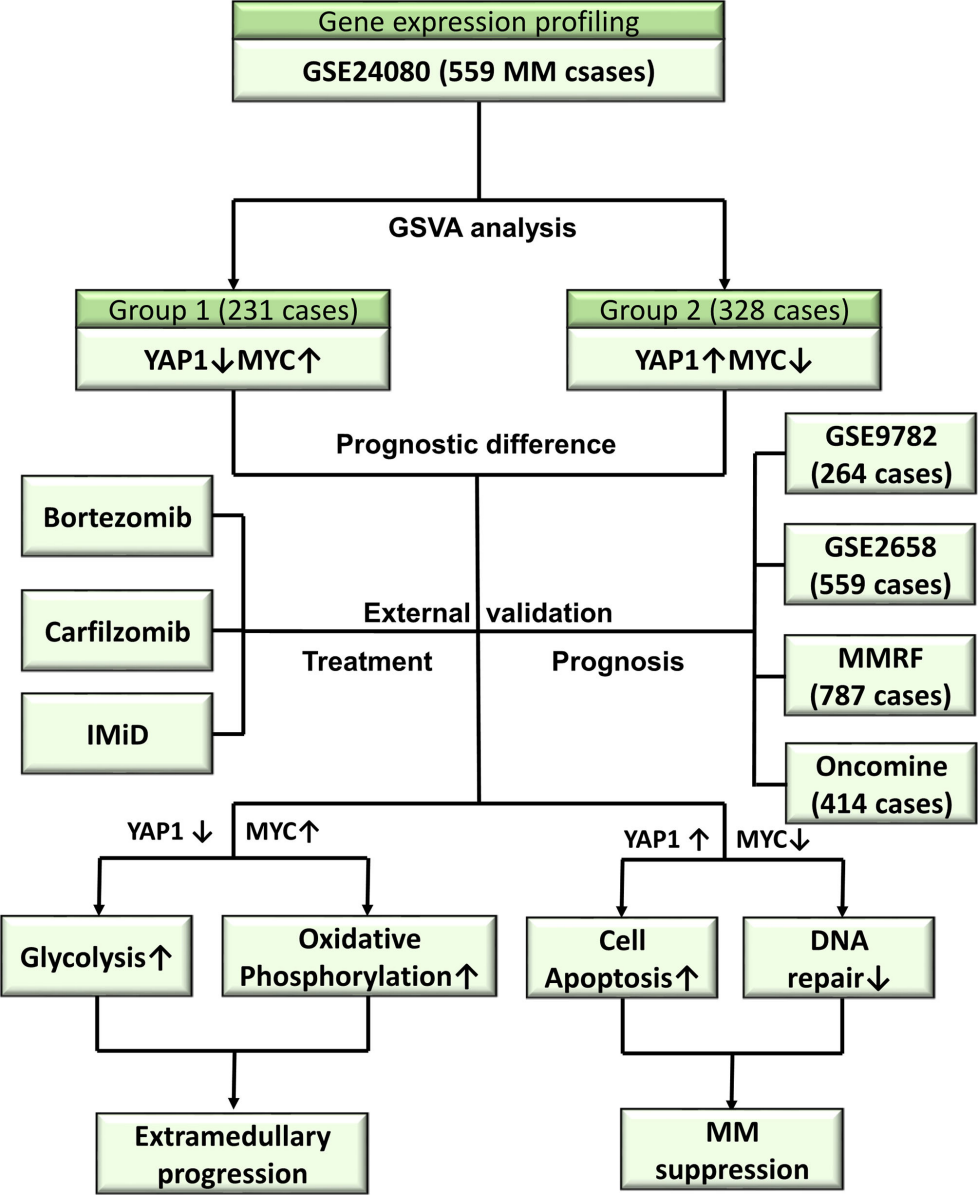
Overview of the study.

From the above results, we concluded that DNA repair/glycolysis/oxidative phosphorylation processes were improved in Group 1, which exhibited enhanced oncogenicity and stronger propensity for extramedullary invasion. We identified that higher activities of these 3 processes were significantly correlated with worse survival in MM (**Figure 7A** and **Figures S7A–C**). An integrated risk score based on the above 3 processes was calculated for each MM patient using the COX regression model (**Figure 7B**). The optimal cutoff value was selected by using the Gordon index (cutoff = 0.21), and KM curves of survival was also performed in **Figure 7C**. The same result was confirmed in the MMRF-COMPASS dataset (**Figure S7D**). Furthermore, we performed a multivariable analysis corrected for ISS stages using the MMRF dataset. The result showed that YAP1 expression and glycolysis activity functioned as prognostic factors even in a multivariable fashion (**Figure S7E**). Also, we observed that enhanced DNA repair and glycolysis activity would impair the therapeutic response of bortezomib-based and bortezomib+carfilzomib-based therapy respectively (**Figures 7D, E**). Interestingly, we discovered that the presence of unfavorable chromosomal alteration such as t(4;14) and t(11;14) also enhanced the activity of DNA repair/glycolysis/oxidative phosphorylation processes (**Figures S7F, G**). The entire study is summarized in **Figure 8**.

## Discussion

YAP1 is a crucial transcriptional coactivator in the Hippo pathway and crosstalks with various cancer-promoting pathways (11). YAP1 facilitates cancer progression in numerous ways, including promoting cell proliferation (12, 13), expansion of cancer stem cells (14, 15), and drug resistance (16, 17). Since the genetic or pharmacologic inhibition of YAP1 suppresses tumor progression and improves drug sensitivity, targeting YAP1 is considered as a novel therapeutic target in various cancers. Immunotherapy has been regarded as a major branch of cancer treatments in recent years. Most recently, YAP1 was reported to act as a contributor in inducing immunosuppressive tumor microenvironment by upregulating programmed cell death ligand 1 (PD-L1) or stimulating tumor cells to recruit tumor-infiltrating macrophages, MDSCs, and Tregs (18–21). However, these findings are mostly made in solid tumors. The expression level of YAP1 is relatively low in hematologic cancers. Previous studies pointed out that the low level of YAP1 in hematologic cancers suppressed the DNA damage-induced apoptosis, facilitating tumor cell survival (9). In MM, extramedullary invasion is regarded as the end stage and lacks therapeutic guidelines. Hence, the role of YAP1 in multiple myeloma progression especially EM needs to be further studied.

In our study, we discovered that a subgroup of MM patients exhibited a specific YAP1-MYC+ phenotype with an unpromising prognosis and a higher recurrence rate. Notable, in solid tumor, YAP1 acts as an upstream transcriptional activator for MYC. Although this study was based on the GSVA analysis of oncogenic gene sets in 559 MM patients, we needed to stress that most *C6-oncogenic signature gene sets* were acquired in solid tumor tissue or cell lines. This might explain the inconsistency that the expression of YAP1 and MYC was negatively correlated in MM, while their downstream molecules according to *C6-oncogenic signature gene sets* were positively related in **Figure 2A**. Additionally, we discovered that YAP1 affected the responsiveness of different treatments in MM. Especially to IMiD, YAP1-MYC+ MM patients exhibited a significantly better outcome, which might be attributed to the immunomodulatory role of YAP1. Previous studies demonstrated the positive regulation of YAP1 in PD-L1 expression (20). Considering the poor efficacy of PD-L1 targeting therapy in MM, YAP1 might serve as a novel immune target. Also, we found that the efficacy of carfilzomib-based treatment was superior to bortezomib-based in any group, consistent with previous reports (22).

Extramedullary invasion is an advanced stage of MM. Currently, there are no guidelines regarding EM treatment. Hence, it is of urgent need to elucidate the molecular mechanism behind EM and identify potential therapeutic targets. We found that in YAP1-MYC+ MM patients, the activity of DNA repair, glycolysis and oxidative phosphorylation was enhanced. This finding was in concordance with a previous report that EM myeloma cells exhibit more active glycolysis and oxidative phosphorylation pathways (10). Also, the expression of YAP1 was even lower in sPCL samples than primary MM samples, suggesting that the low expression of YAP1 in MM might accelerate extramedullary invasion. We also confirmed our findings on the single-cell level for which the sc-seq transcriptome data of 477 MM and EM myeloma cells were acquired. The trajectory analysis revealed that MM myeloma cells experienced a transcriptomic evolution to transform into EM myeloma cells and the expression of glycolysis and oxidative phosphorylation genes were significantly stronger in EM myeloma cells. However, due to the low expression level, the single-cell data lack YAP1. Also, the nature of our study is a retrospective *in silico* analysis that has certain limitations. Hence, the underlying molecular mechanisms need to be further explored using *in vitro* and *in vivo* experiments and statistically powered studies for hypothesis validation. Furthermore, the accumulation of the YAP/TAZ complex led to BRAF inhibitor resistance *via* increasing actin remodeling in melanoma (23), while in MM, BRAFV600E mutation was widely detected and posed as a druggable target in extramedullary invasion especially in the central nervous system (24), which indicated that further studies should be performed to investigate the predictive ability of YAP1 in BRAF inhibitor sensitivity in EM cases.

Collectively, we performed a multi-center, retrospective integrated transcriptomic analysis on both bulk and single-cell level in MM. We discovered a distinctive group of MM patients exhibiting YAP-MYC+ phenotype and worse outcomes. These patients showed high responsiveness to IMiD treatment. The improved oncogenicity might be attributed to enhanced activities of DNA repair, glycolysis, and oxidative phosphorylation, which might accelerate extramedullary invasion.

## Data Availability Statement

The original contributions presented in the study are included in the article/**Supplementary Material**. Further inquiries can be directed to the corresponding author.

## Ethics Statement

Written informed consent was obtained from the individual(s) for the publication of any potentially identifiable images or data included in this article.

## Author Contributions

BZ and RL designed the study. WS, KY, YZ, LW, HL, CZ, and HX collected transcriptome data. BZ analyzed the data and wrote the paper. All authors contributed to the article and approved the submitted version.

## Funding

This work was supported by the National Natural Science Foundation of China (No. 81870159) and the Shanghai Pujiang Talent Program (No.18PJD059).

## Conflict of Interest

The authors declare that the research was conducted in the absence of any commercial or financial relationships that could be construed as a potential conflict of interest.

## Publisher’s Note

All claims expressed in this article are solely those of the authors and do not necessarily represent those of their affiliated organizations, or those of the publisher, the editors and the reviewers. Any product that may be evaluated in this article, or claim that may be made by its manufacturer, is not guaranteed or endorsed by the publisher.
