# Supplementary material for: Integrated Transcriptomic Analysis Reveals a Distinctive Role of YAP1 in Extramedullary Invasion and Therapeutic Sensitivity of Multiple Myeloma
Source: Front Oncol. 2022 Jan 4;11:787814. doi: 10.3389/fonc.2021.787814 (PMC8763979; doi:10.3389/fonc.2021.787814)
Supplement: Supplementary file 2 [file DataSheet_2.docx]

**List**

**Supplementary figure 1**

**Supplementary figure 2**

**Supplementary figure 3**

**Supplementary figure 4**

**Supplementary figure 5**

**Supplementary figure 6**

**Supplementary figure 7**

**
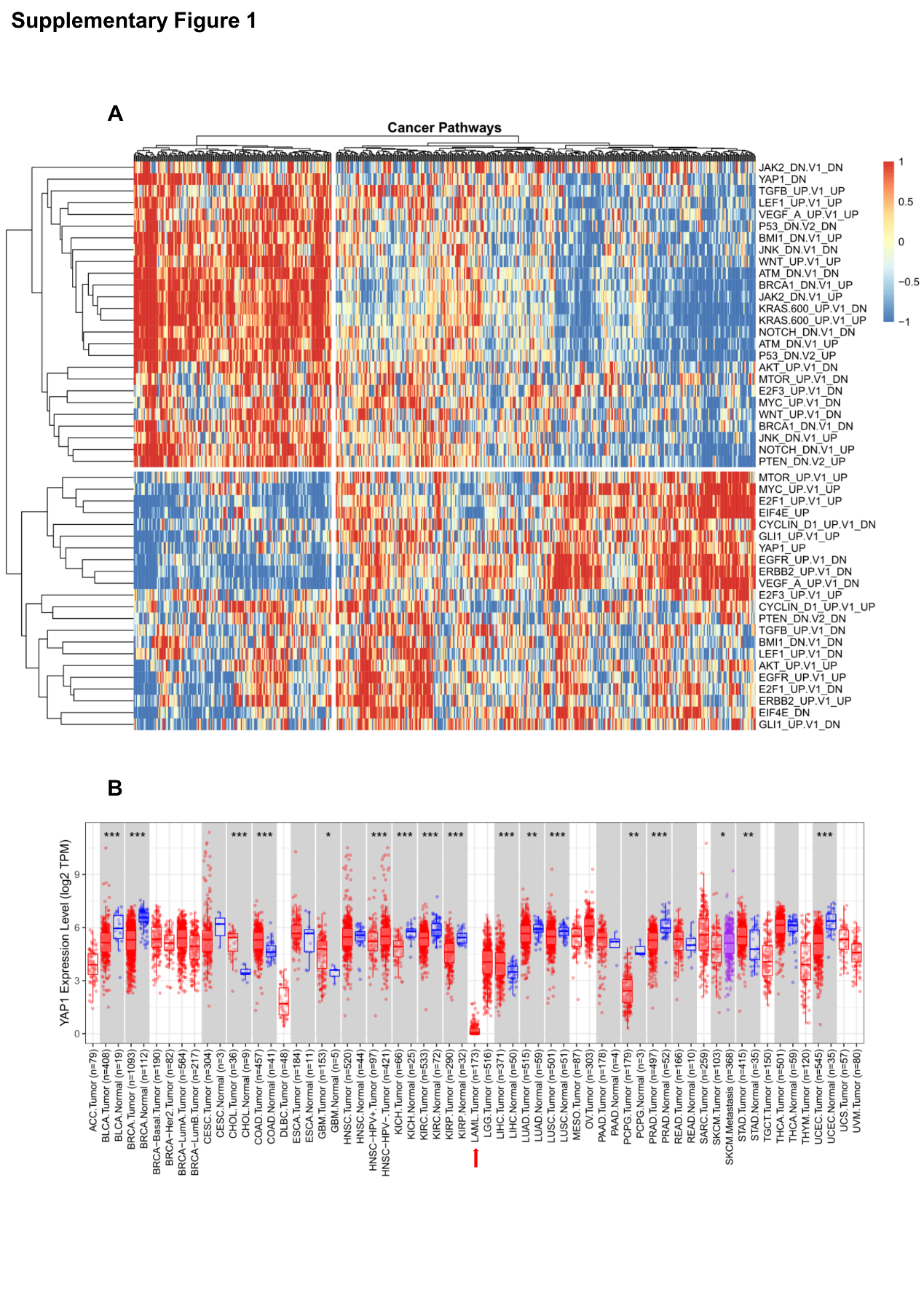
**

**Figure S1.** A. Heatmap of gene set variation analysis (GSVA) of 559 Multiple Myeloma transcriptome data (GSE24080) based on all *C6-oncogenic signature gene sets* from Molecular Signature Database. B. YAP1 expression of different tumor types in the TIMER database. Data are presented as mean ± SD. *P<0.05; **P<0.01;***P<0.001.

**
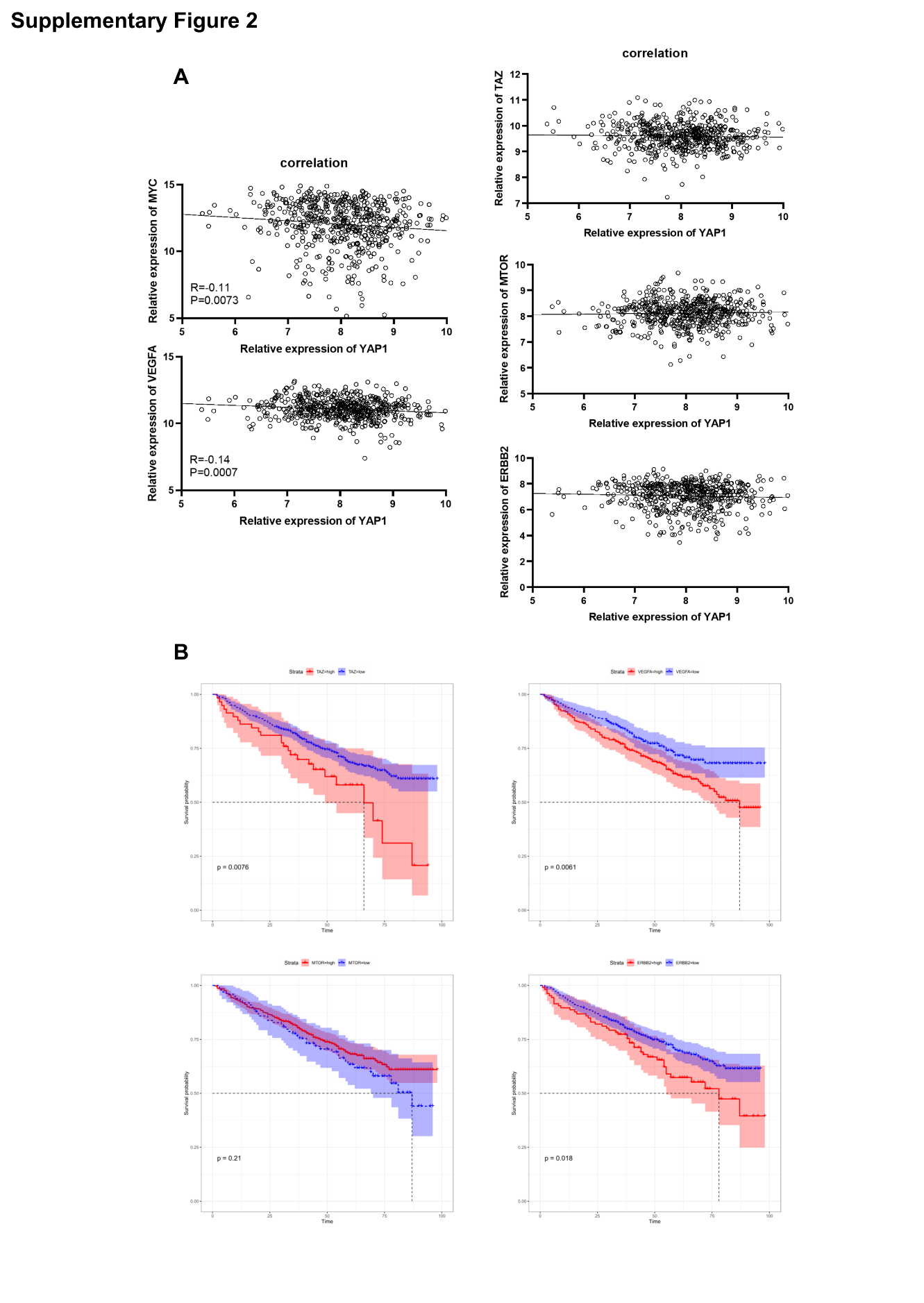
**

**Figure S2** A. The correlation of the mRNA expression level between YAP1 and MYC/TAZ/MTOR/ERBB2/VEGFA using Pearson correlation coefficient. B. Kaplan–Meier survival curves comparing the overall survival in MM cases with high or low expression of TAZ/VEGFA/MTOR/ERBB2 in GSE24080 dataset.

**
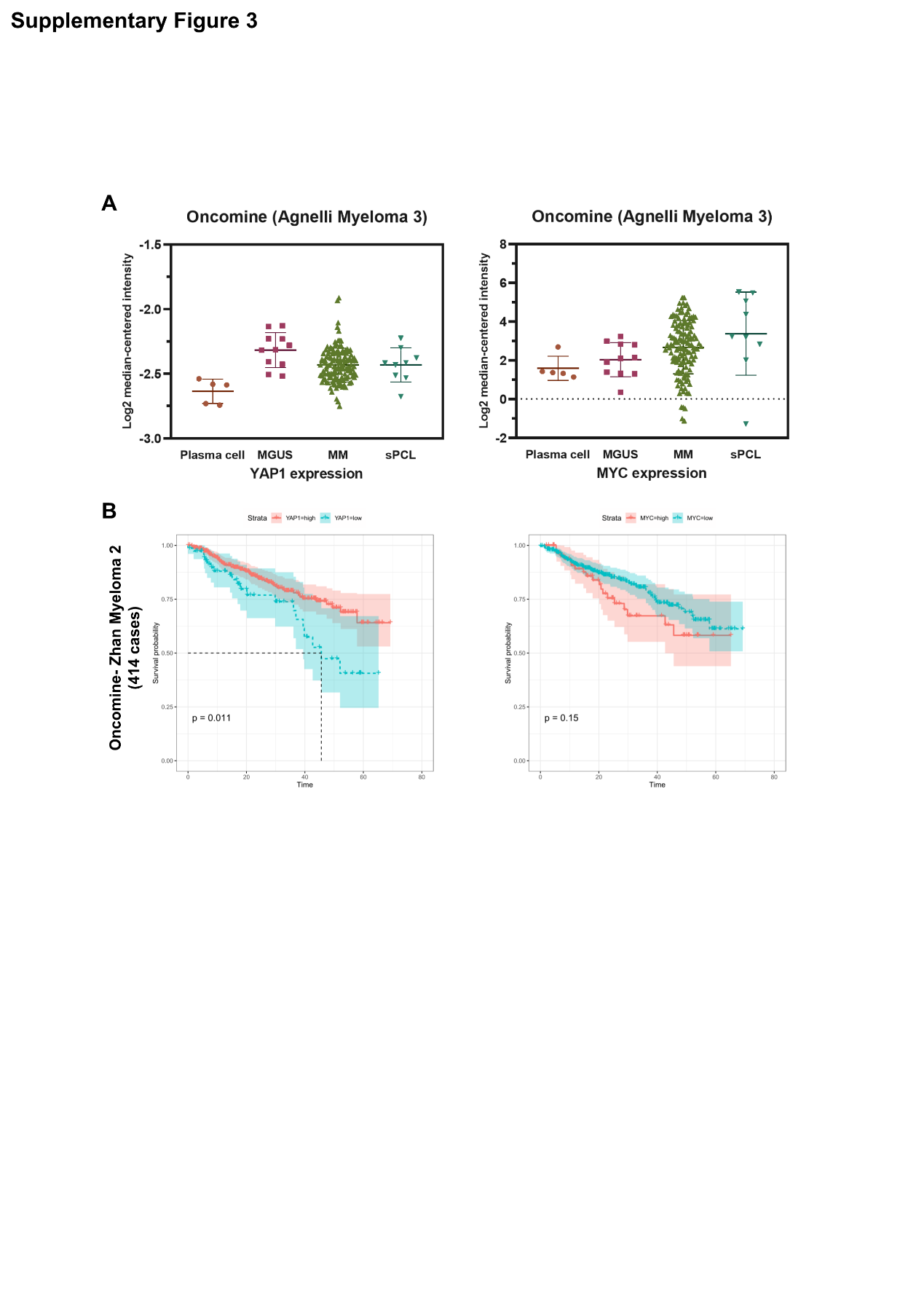
**

**
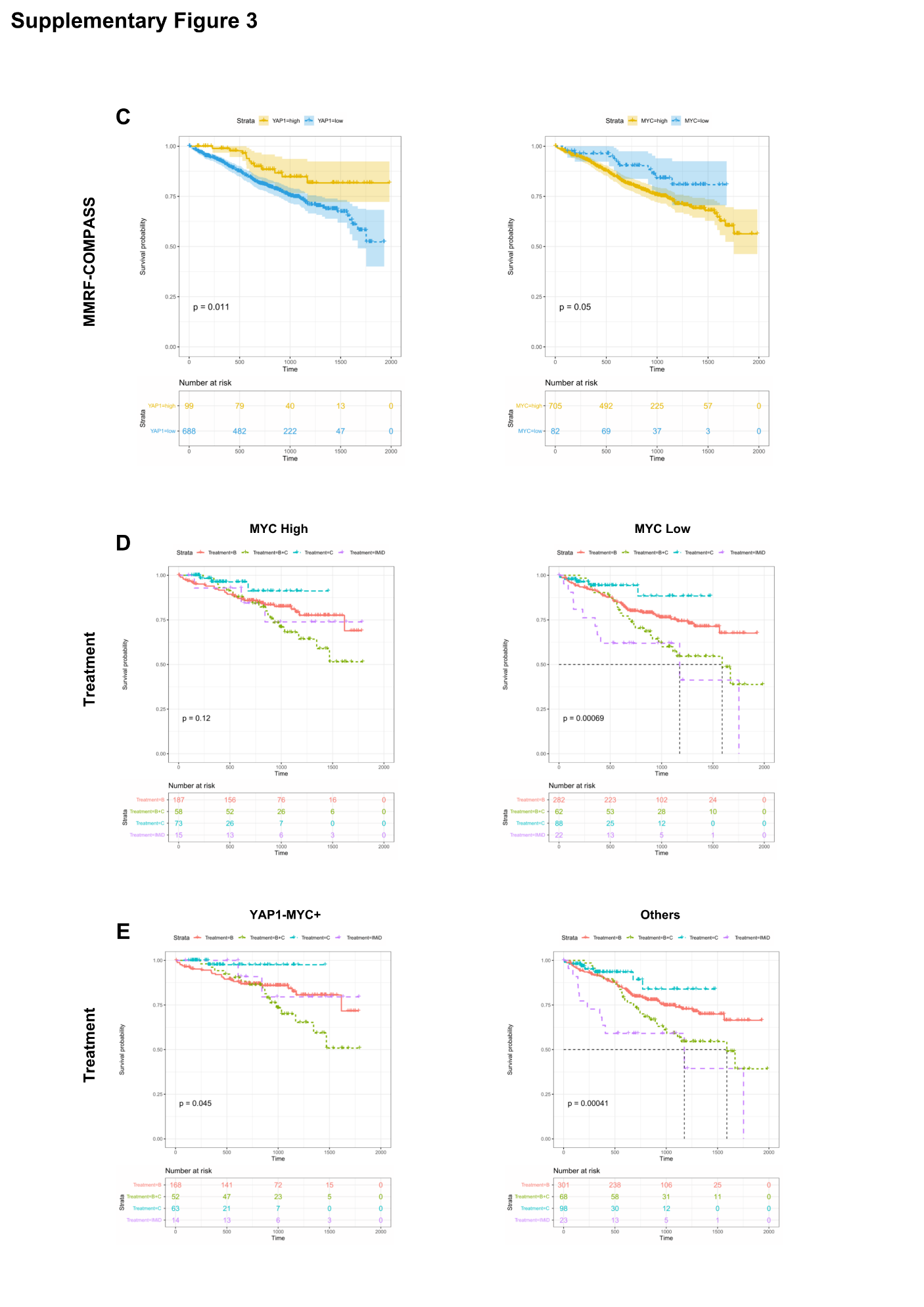
**

**Figure S3** A. The expression level of YAP1/MYC in different developmental stages of MM based on the data from Oncomine. B. Kaplan–Meier survival curves comparing the overall survival in MM cases with differential expression level of YAP1 and MYC in Oncomine dataset (414 cases). C. Kaplan–Meier survival curves comparing the overall survival in MM cases with differential expression level of YAP1 and MYC in MMRF-COMPASS dataset (787 cases). D. The survival rate of MM patients with high or low expression of MYC treated with different strategies. E. The survival rate of YAP1-MYC+ MM patients versus other MM patients treated with different strategies.

**
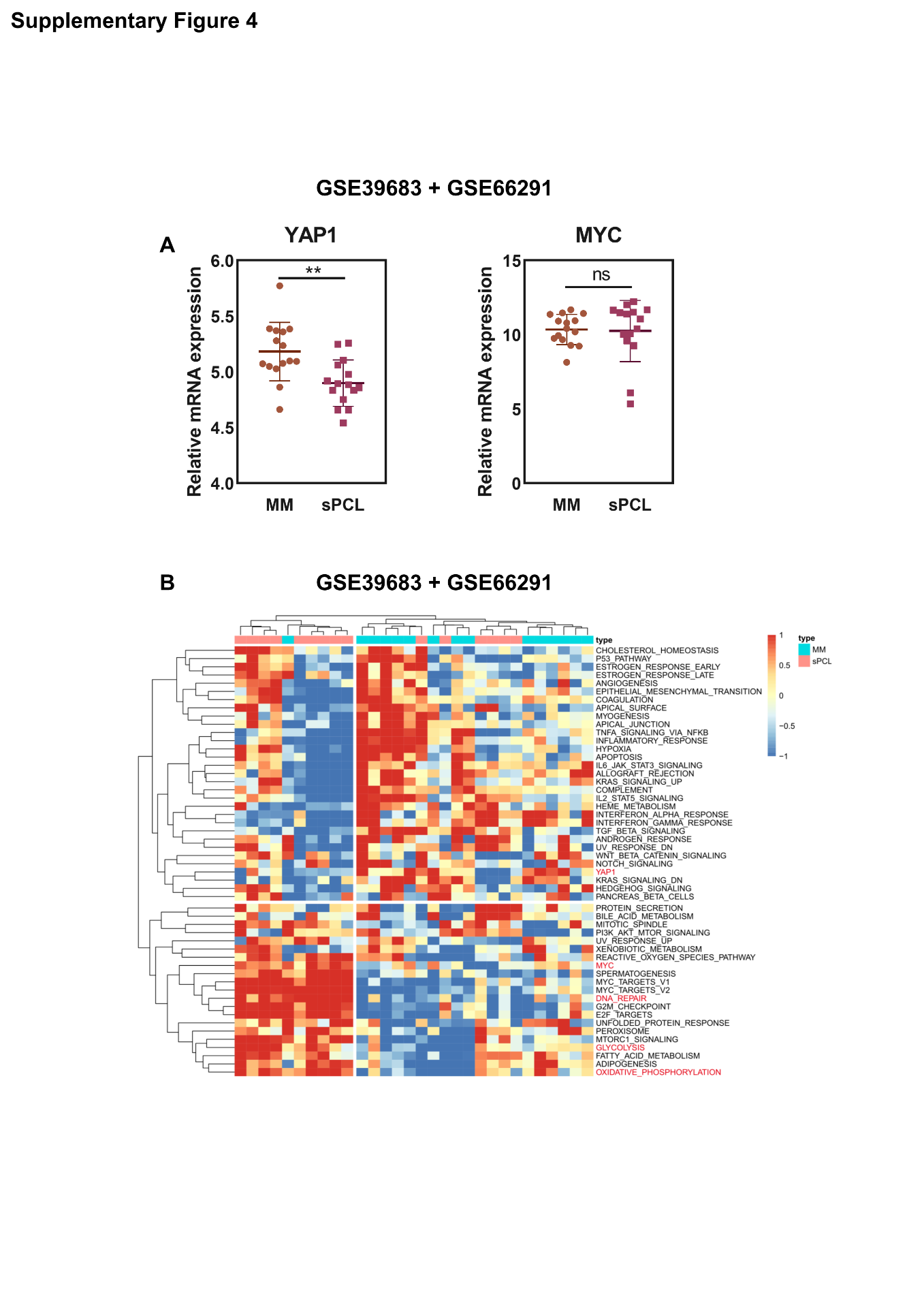
**

**Figure S4** A. The comparison of the expression level of YAP1/MYC between primary MM and secondary plasma leukemia based on GSE39683 and GSE66291 datasets. Data are presented as mean ± SD. *P<0.05; **P<0.01;***P<0.001. B. GSVA analysis of primary and secondary plasma leukemia based on GSE39683 and GSE66291 datasets.

**
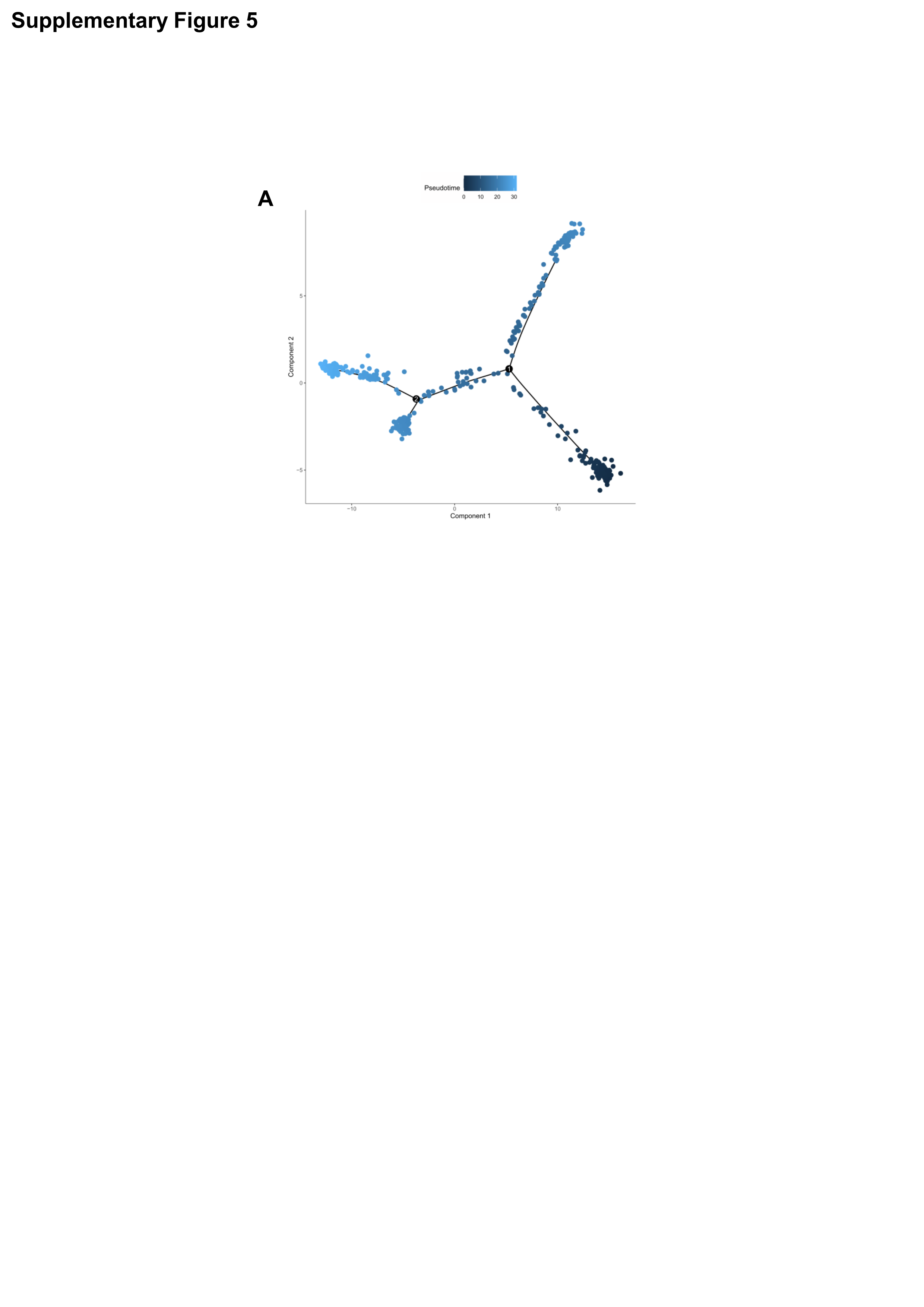
**

**Figure S5** A. Pseudo time analysis of 447 single-cell transcriptome data via Monocle algorithm. Pseudo timeline was color-coded.

**
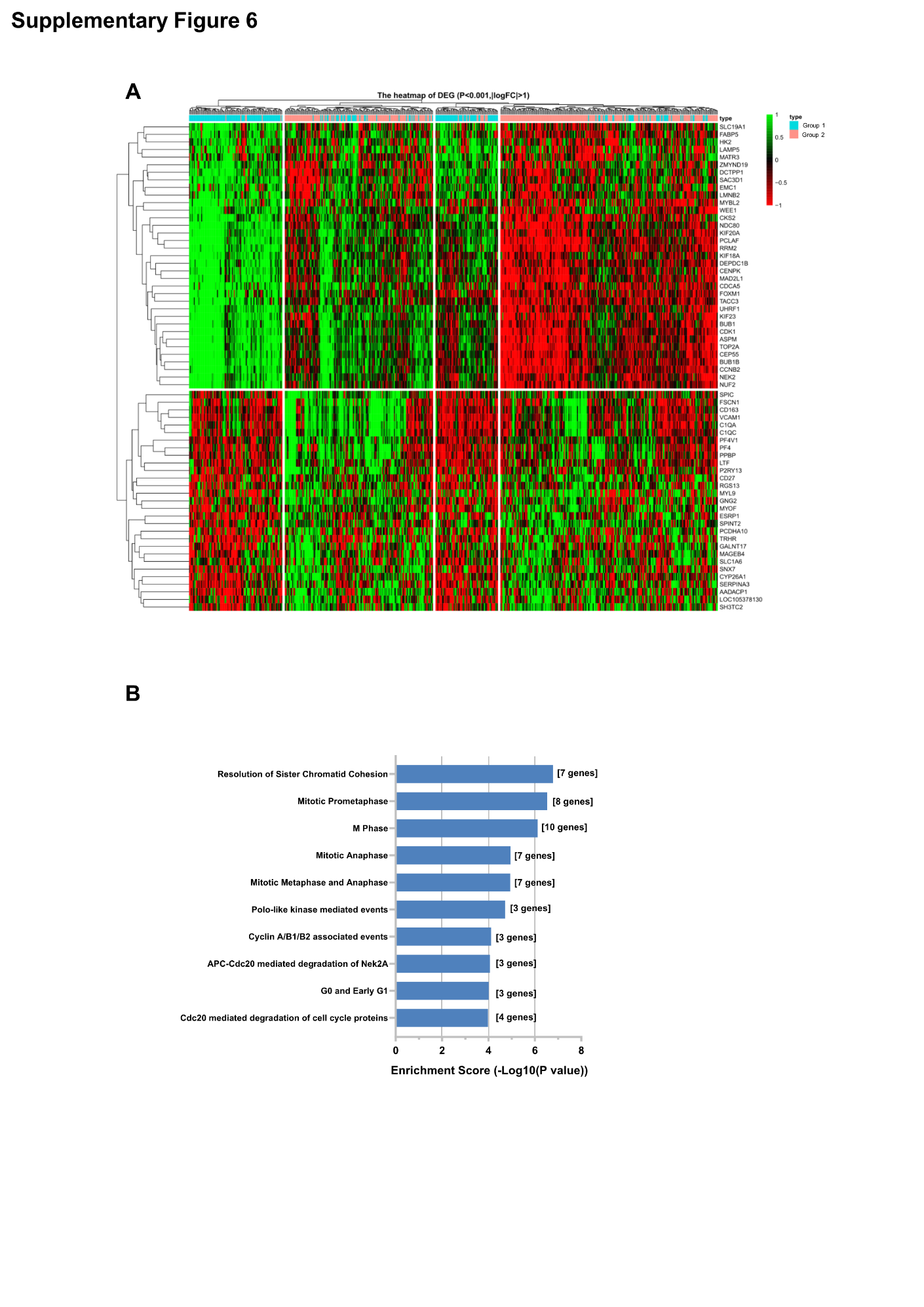
**

**
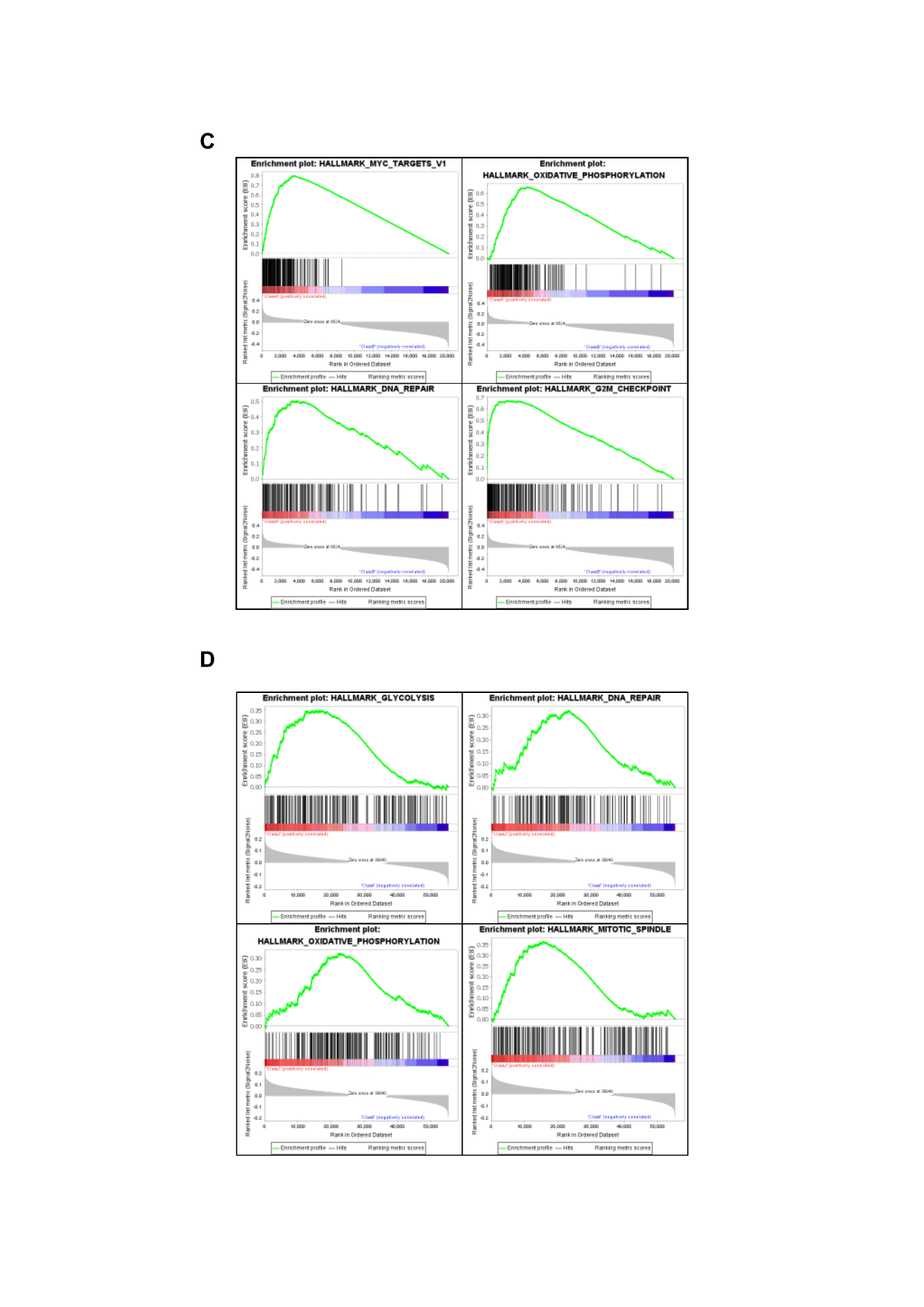
**

**
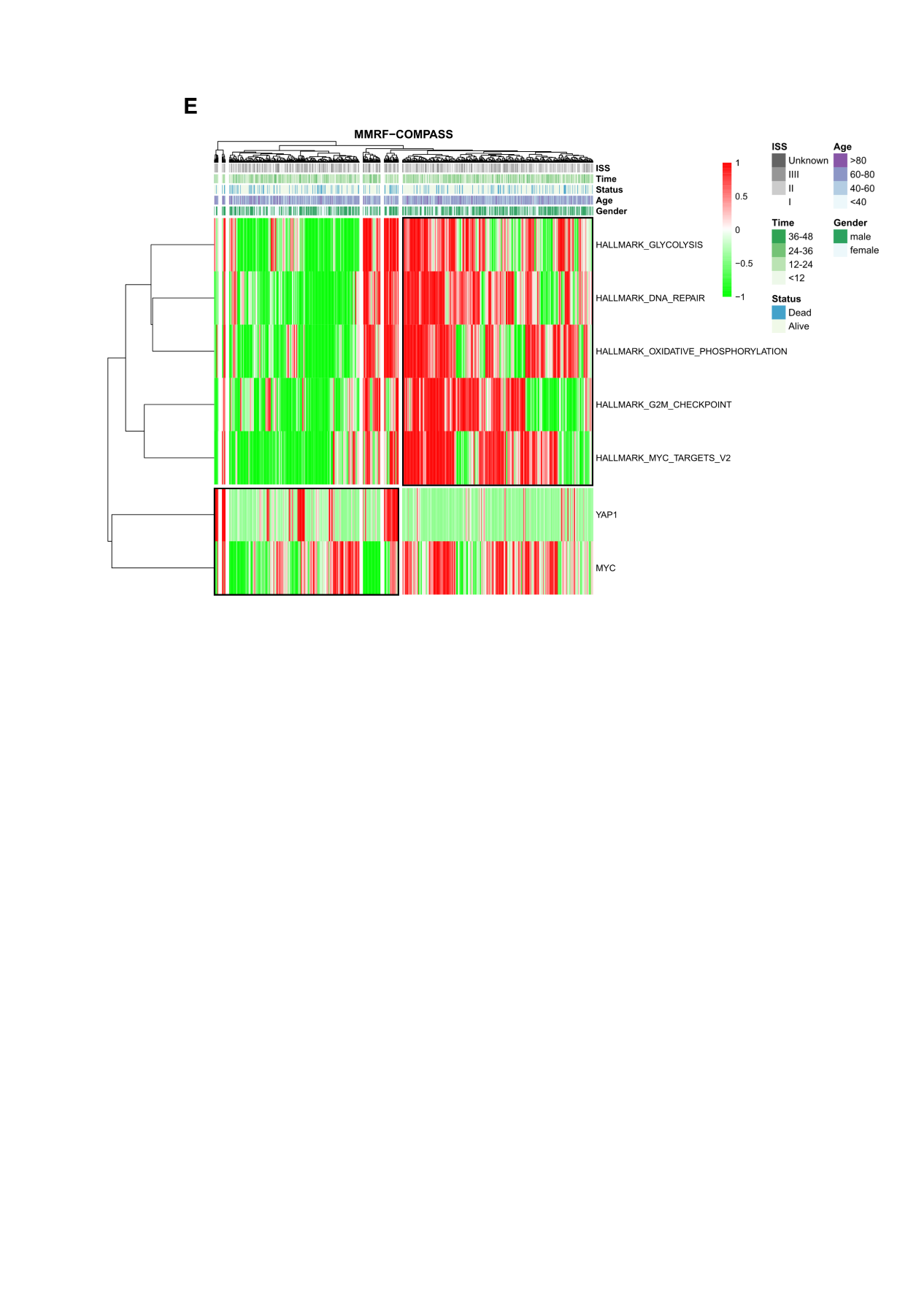
**

**Figure S6** A. Heatmap of DEGs (p<0.001, |log2FC|>1) of group 1 relative to group 2 MM samples. B. Reactome pathway enrichment analysis by the “Clusterprofiler” package of R language. The number of affected genes in each pathway is shown. The statistical significance of the enrichment is expressed as -log10(P value). C. The GSEA analysis of Hallmark pathways upregulated in group 1 MM patients. D. The GSEA analysis of Hallmark pathways upregulated in MMRF patients with the presence of soft tissue plasmacytoma. E. GSVA analysis of key gene sets along with YAP1/MYC expression in MMRF cohort.

**
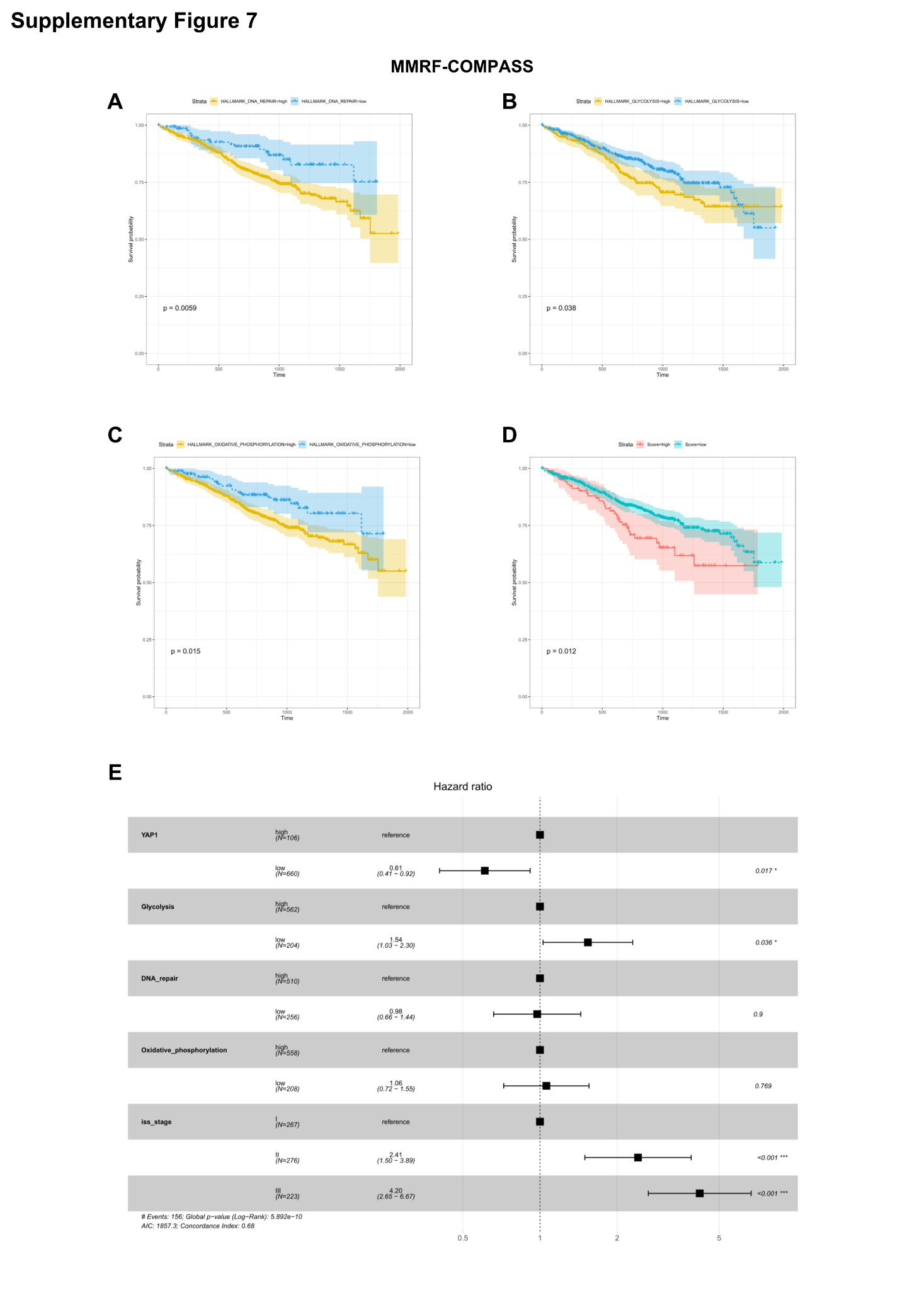
**

**
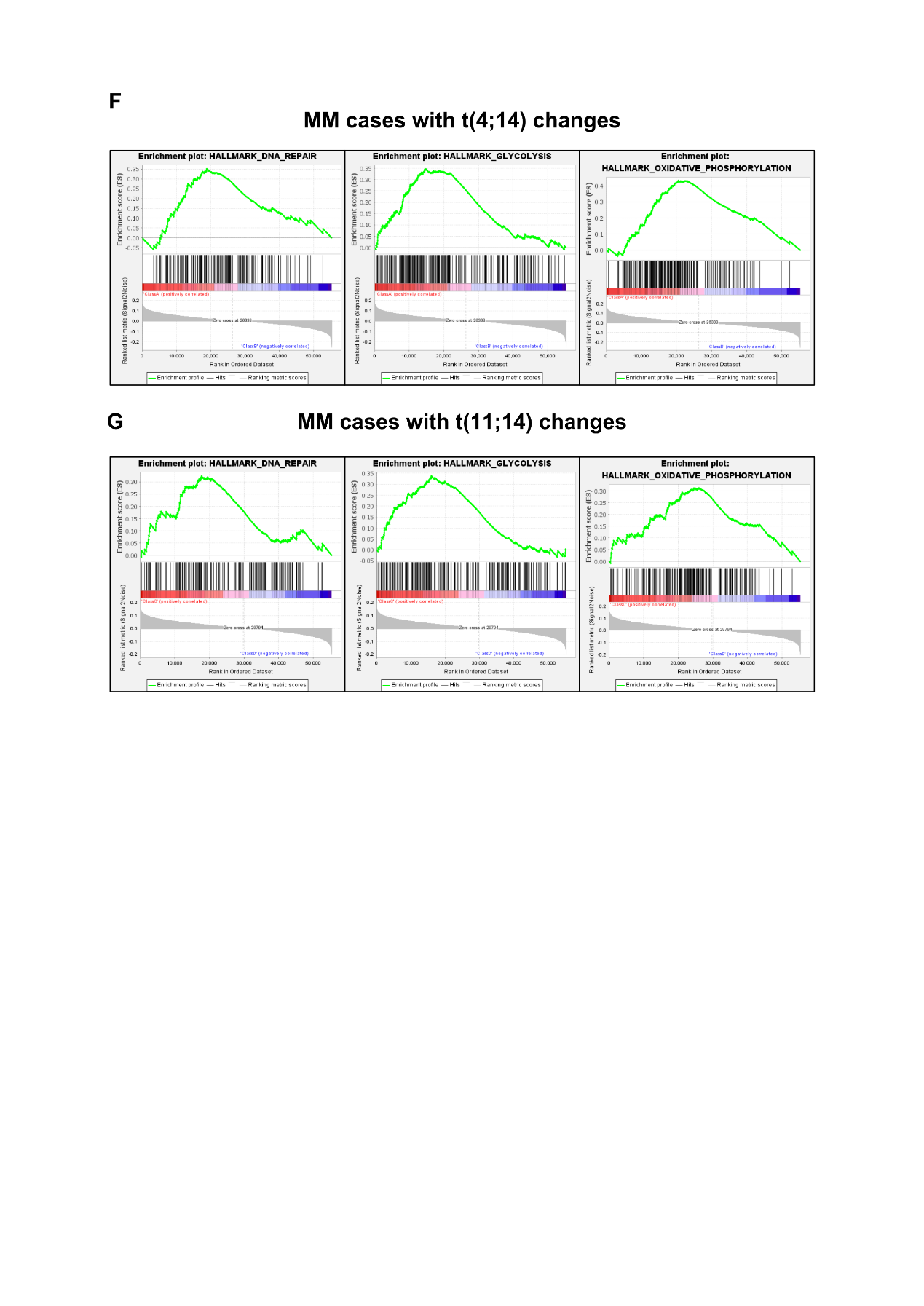
**

**Figure S7** A-C. Kaplan–Meier survival curves comparing the overall survival in MM cases with high or low expression of DNA repair/Glycolysis/Oxidative phosphorylation scores in MMRF-COMPASS dataset. D. Kaplan–Meier survival curves comparing the overall survival in MM cases with high or low risk score in MMRF-COMPASS dataset. E. Forest plot showed the multivariate result of YAP1, activities of DNA repair/Glycolysis/Oxidative phosphorylation pathways along with ISS stage in MM. F. GSEA analysis of MMRF MM cases with/without t(4;14). G. GSEA analysis of MMRF MM cases with/without t(11;14).
